# Comprehensive Indicators for Evaluating and Seeking Elasto-Magnetic Parameters for High-Performance Cable Force Monitoring

**DOI:** 10.3390/s22207776

**Published:** 2022-10-13

**Authors:** Shuangsheng Yan, Yujue Wang, Peng Li, Zhichao Gao, Bin Wu, Xiucheng Liu

**Affiliations:** Faculty of Materials and Manufacturing, Beijing University of Technology, Beijing 100124, China

**Keywords:** elasto-magnetic effect, cable force measurement, comprehensive indicators, performance evaluation

## Abstract

The elasto-magnetic method is a promising pathway for cable force monitoring in cable-stayed bridges. Under the action of an externally applied pulsed magnetic field, both the variation in the main flux recorded by the induction coil and the localized surface magnetic field measured by the packaged magnetic sensor are typical signals for observing the elasto-magnetic effect in tensioned cables. However, the performances of the parameters extracted from the two types of elasto-magnetic signals are never strictly compared in the experiment. Meanwhile, comprehensive indicators for evaluating the ability of elasto-magnetic parameters on cable force characterization are seldom discussed. As a result, it is difficult to compare the performances of elasto-magnetic devices developed by different teams, and the pathway of seeking new parameters for cable force monitoring is obstructed. In this study, elasto-magnetic calibration experiments were performed on a cable of seven-wire steel strands to simultaneously measure the variation in the main flux and the localized surface magnetic field. Comprehensive indicators considering sensitivity, hysteresis error, and cable force resolution are proposed to examine the performances of classic elasto-magnetic parameters and new candidate ones. Through comparative study, two new parameters demonstrated outstanding ability for cable force measurement, and they are the minimum amplitude of the induced voltage and the area under the curve between two points of 3 dB height of the voltage measured by a Hall sensor. The latter is recommended for high-performance cable force monitoring from the perspective of simplicity in sensor configuration.

## 1. Introduction

Cable-stayed bridges are structures in which the main deck is directly suspended by a large number of steel cables connected to the tower. Each cable bears tensile force, and the cable force is one of the key indicators for evaluating the health state of the cable-stayed bridge during its construction and in-service stages. Once the cable force exceeds or falls below its expected threshold, the force balance system of the cable-stayed bridge may suffer a rising risk of collapse caused by structural failure. Therefore, the cable force measurement is essential for examining and monitoring the health status of cable-stayed bridges.

Among the available pathways for cable force measurement [1,2,3], the elasto-magnetic (EM) method is widely used for cable force monitoring due to the simple structure and good durability of the EM sensor [4]. The stress inside the ferromagnetic materials alters the magnetism of the material subjected to the externally applied magnetic field. The variation in magnetic features such as the change in stress is referred to as the elasto-magnetic effect. The EM effect can be experimentally observed in non-contact ways, utilizing the nature of the magnetic field [5]. Coil sensors are commonly employed for EM effect monitoring, and the sensor can be wound onto the cable on site, facilitating it to be more applicable for the cables’ in-service as compared with the stress gauge that must be pre-installed at the construction stage [6]. Moreover, the EM effect is mainly related to the magnetization behavior of the material and independent of the length of the cable. Therefore, the calibrated EM sensor is feasible for monitoring the cable force with different lengths. This would be a significant advantage of the EM method over the vibration-frequency-based cable force measurement method [7].

Numerous studies have proven that the EM method is a promising pathway for evaluating the tensile force in steel strands and steel stay-cables [8,9,10]. The earliest EM sensors were made by two coaxial solenoid coils. Pulse current with high amplitude was generated by various types of charging–discharging circuits and then fed into the primary coil of the EM sensor to provide a pulsed magnetic field. Under the action of the pulsed magnetic field, the strands/cables would experience rapid magnetization approaching their near-saturation state. A secondary coil was used for measuring the variation of the main flux in the cable. The peak amplitude of the induced voltage in the secondary coil was proposed by Wang et al. in the 1960s for cable force characterization [11,12]. Since then, researchers have never stopped innovating and improving EM technology for cable-stayed bridges. EM sensors with improved configuration and new magnetic sensing units are proposed to seek new magnetic features for realizing high-performance cable force characterization.

Kim et al. [13] developed an embedded coil-based EM sensor to monitor the tensile force of pre-stressed tendons used in the concrete girders of bridges. Field test results demonstrated success in the linear characteristic of tensile force with the area of the B-H loop. The authors of this study attempted to find new parameters for cable force measurement from the spectrum of the induced voltage in the secondary coil. The summation of the spectrum amplitudes in a particular band demonstrated superior performances in accurately characterizing the cable force over the conventional time-domain parameters [14]. Duan et al. [15,16,17] made a breakthrough in applications of the EM method for in-service cable force monitoring by replacing the secondary coil with a magnetic sensor of TD/PMNT/TD-laminated composites. The small-size magnetic sensor of the laminated composites was used to measure the localized magnetic field near the cable surface (not the main flux in the cable) as the voltage signal during the cable magnetization process. The utilization of a small-size magnetic sensor made the structure of the coil sensor more concise. Ref. [16] claimed that the magnetic sensor of TD/PMNT/TD-laminated composites has the advantage of high sensitivity as compared with the conventional Hall sensor in EM applications. It should be pointed out that the description of high sensitivity was correct only for magnetic field measurement. The sensitivity of parameters extracted from the EM signal to the cable force is not in proportional correlation with the sensitivity of the magnetic sensor in magnetic field measurement.

Under the pulse magnetization mode, the peak strength of the applied magnetic field is high enough to induce a voltage signal of a high signal-to-noise ratio in a magnetic sensor, including the conventional Hall sensor and the induction coil. The key point to estimate the performance of the EM sensor is to evaluate the ability of EM parameters in the quantitative characterization of cable force, including the linearity, sensitivity and hysteresis characteristics, etc. According to the mechanism of the EM effect, the entire magnetization process of the steel strand/cable is affected by the externally applied tensile force. As a result, plenty of parameters, which are sensitive to the variation in tensile force, can be extracted from the measured EM signals whether it was obtained by a coil or a magnetic sensor [18]. The calibration results denoting the relationship between the parameters of the EM signal and the cable force highly depend on the used EM sensor and instrument. Thus, the performances of EM parameters reported by different researchers cannot be directly compared. To solve this limitation, a comprehensive indicator describing the performances of EM parameters in cable force estimation is required.

In this study, EM calibration experiments were performed on a cable composed of a bundle of seven-wire steel strands using a coil sensor wound by multi-conductor electric cables and a self-developed miniaturized EM device. The process of cable magnetization is demonstrated as the excitation pulse current fed into the primary coil, the voltage induced in the secondary coil, and the output signal of the Hall sensor placed near the cable surface. In such a way, plenty of EM parameters can be synchronously measured, and their performances in cable force characterization can be compared under the same conditions. A comprehensive indicator combining sensitivity, hysteresis error, and cable force resolution was proposed to evaluate the performance of EM parameters. Among the investigated EM parameters, the frequently used ones extracted from the induced voltage demonstrate average ability in cable force characterization. Through comparative analysis, two new parameters that have not been reported yet are found to possess optimal ability in cable force measurements.

The rest of this article is organized as follows. In Section 2, the experimental set-up, including the EM device, EM sensor, and cable tension system, is described. The EM signal processing method and the investigated parameters of various types of EM signals are discussed in Section 3. The performances of the EM parameters on cable force characterization are evaluated in Section 4. Finally, the findings of this work are summarized in Section 5.

## 2. Experimental Set-Up

The accurate relationship between magnetic features and tensile force is difficult to predict in theory due to the absence of key factors related to the structural parameters of the EM sensor and the configuration of strands or cables. Therefore, calibration of the EM sensors and devices was required to perform before applications. The basic calibration procedure for the EM method includes magnetic field excitation accompanied by magnetic response measurement, feature extraction for tensile force characterization, and determination of calibration equations.

**Figure 1:**
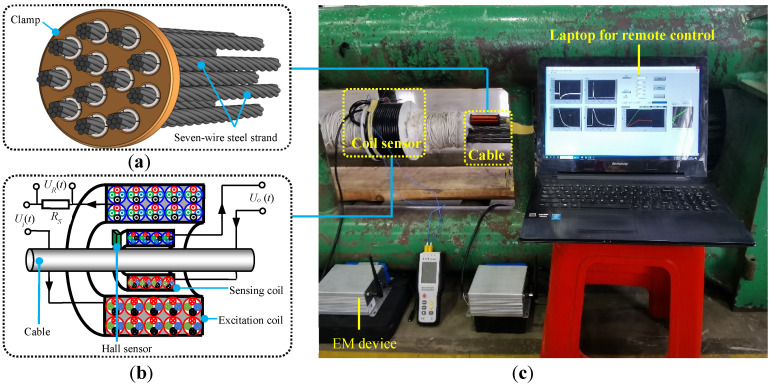
Experimental set-up for calibration: (**a**) cable composed of a bundle of seven-wire steel strands, (**b**) coil sensor, and (**c**) experimental set-up.

An EM calibration system was constructed on a platform of a cable tension machine. The entire experimental set-up is shown in Figure 1, employing a coil sensor wound by multi-conductor electric cables and a self-developed miniaturized EM device. A cable composed of a bundle of seven-wire steel strands with a length of around 6 m was used for the tension test. The configuration of the cable is shown in Figure 1a. A total of 12 seven-wire steel strands were clamped by an anchor disk. The nominal outer diameters of a single strand and the entire cable are about 15.2 mm and 95 mm, respectively. Both the tension machine and the tested cable were provided by Liuzhou OVM Engineering Co., Ltd., China. The expected breaking force of the cable is around 2860 kN. To suppress the effect of bending caused by the weight of the steel cable on the measured magnetic responses, the cable was pre-tensioned to 200 kN before EM calibration considering that the normal load of the cable in-service is around 1144 kN. The cable experienced step-by-step load and unload cycles in the range of 200–1800 kN.

For a given stay-cable, the form of the calibration equation and its constant coefficients highly depend on the EM devices. To realize near-saturation magnetization of internal wires in large-diameter cables, the pulsed magnetic field of high peak strength is preferred in the design of EM devices. The high-voltage charging–discharging circuit and the coil sensor is the core of the pulsed magnetic field excitation component. Coil sensors that can be conveniently wound on site would greatly facilitate the cable force monitoring for in-service bridges. Conventional coil sensors are wound by single-core enameled copper wire. It is very time-consuming when the number of winding turns is large. To save time and consider the convenience of field testing, multi-conductor electric cables are employed for coil sensor winding. The number of winding times is equal to the required total number of coil turns divided by the number of conductors in a single electric cable. For instance, in the experiment, an electric cable with twelve conductors was used for winding the excitation coil, and the winding time was expected to be one-twelfth of that of the conventional way. After winding the multi-conductor electric cable onto the tested cable, the wire terminals at both ends are connected by a circuit board so that a solenoid coil can be formed to act as an excitation coil or a sensing coil.

The parameters of the excitation coil and sensing coil used in this study are listed in Table 1. The excitation coil was connected with a current-sampling resistor of 0.2 Ω in series and then connected with the output terminal of the EM device. The sensing coil was used to measure the variation of the main flux in the cable. To seek new magnetic features for cable force characterization, the tangential component of the surface magnetic field was simultaneously recorded in the process of main flux measurement. The Hall sensor of CYSJ362A (Chenyang Technology Co., Ltd., Munich, Germany), which has a large linear range of ±2 T and a sensitivity of 3.5 mV/mT under the condition of supply voltage as ±5 V, was employed to measure the surface magnetic field. The technical parameters of the Hall sensor are listed in Table 1. An instrument amplifier of AD620 (Analog Devices, Inc., USA) was used to realize bias voltage removal and differential amplification (6 dB) of the output voltages of the Hall sensor.

**Figure 2:**
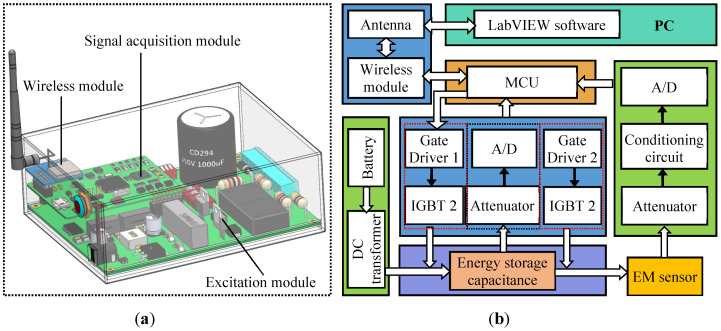
(**a**) Three-dimensional (3D) assembly drawing and (**b**) frame diagram of the developed EM device.

To meet the demand for remote and wireless monitoring of tensile force in cables, miniaturized EM devices of low power consumption are the first choice. The EM device used in the calibration experiment was developed by us, and its configuration is shown in Figure 2. A lithium-ion battery is used to supply power to the EM device, which can be controlled remotely through a wireless network. The whole device is 180 × 130 × 80 mm^3^ in dimensions and weighs around 1 kg. The advantages of light weight, small size, and remote controllability of the developed EM devices are conducive to field applications.

The miniaturized EM device mainly includes an excitation module, a signal acquisition module, and a wireless module. Charging–discharging circuit is the core of the excitation module. An energy-storage capacitor (*C* = 1000 μF, withstand voltage of 450 V, breakdown voltage of 500 V) and an RLC circuit working under the over-damped, non-oscillatory state are constructed for charging and pulse discharge, respectively. Detailed operation principles of the charging–discharging circuit can be found in our previously reported results in Ref. [18]. The charging and discharging process can be switched by controlling the state of two IGBTs (type: IXYX120N120C3, IXYS Corporation, Milpitas, CA, USA) in the circuit. The withstand voltage and current of the selected IGBTs are 1200 V and 60 A, respectively. The state of IGBTs is determined by the two channels of I/O signals issued by a micro-control unit (MCU) of STM32H7 equipped with ARM Cortex-M7.

In the charging circuit, a high-voltage module powered by a 24 V lithium-ion battery stably outputs a DC voltage of 400 V to charge the aluminum electrolytic capacitor. The voltage across the energy storage capacitor is measured by a voltage feedback circuit in real time, and the state of IGBTs is switched for discharging once the voltage across the energy storage capacitor reaches its expected value. Pulse with a peak voltage in the range of 0–400 V (or peak current lower than 30 A) can be generated by the discharging circuit. During the EM calibration tests, pulse voltage with a peak amplitude of 400 V is selected and fed into the excitation coil. Under the action of the pulse magnetic field provided by the excitation coil, the cable would be magnetized. Three types of signals are acquired by the signal acquisition module, and they are excitation current, output voltage of the sensing coil, and the Hall sensor. The sampling rate for the four types of signals is about 100 kS/s. The excitation current, *I*_e_(*t*), is determined by measuring the voltage of a resistance (*R*_s_ = 0.2 Ω), *U_R_*(*t*), connected in series with the excitation coil. Due to the high change rate of the main flux in the cable, the voltage induced in the sensing coil may be higher than 100 V. To protect the acquisition channel and fully capture the waveform of the induced voltage, an attenuator of −29.8 dB is connected between the sensing coil and the acquisition circuit so that the induced voltage can be attenuated to be lower than 10 V. A serial port-to-WiFi module is employed to construct the wireless transceiver circuit. The WiFi module embedded into the EM device acts as the TCP server, and a laptop is used as a TCP client to remotely control the EM device and receive the data of the acquired signals.

## 3. Signal Processing and Feature Extraction

Under the action of the pulsed magnetic field, the cable experienced a complex magnetization process. Its magnetization trajectory in the B-H chart starts from the initial point of the demagnetization state and rapidly reaches the near-saturation region, followed by a return to the remanence point. For a given cable, the shape of its magnetization trajectory is mainly determined by the peak amplitude, duration, change rate of the rising–falling edges, etc., of the pulsed magnetic field. The strength of the pulsed magnetic field provided by the excitation coil can be roughly estimated as *H*(*t*)= *N*_1_*U_R_*(*t*)/*R*_s_, where *N*_1_ represents the number of coil turns in the primary coil. Digital integration of the voltage signal induced in the secondary coil, *U_o_*(*t*), in a magnetization period *T* can be used to calculate the magnetic induction intensity *B*(*t*) in the cable [18]:

(1) $$B\left(t\right)=\int_{0}^{T}\frac{U_{o}\left(t\right)}{N_{2}A_{s}}dt+\mu_{0}\left(1-\frac{A_{m}}{A_{s}}\right)H(t)$$

where *N*_2_ and *A_m_* are the turns and cross-sectional area of the secondary coil, respectively; *A*_s_ is the sectional area of the tested steel cable; *μ*_0_ is the vacuum permeability.

**Figure 3:**
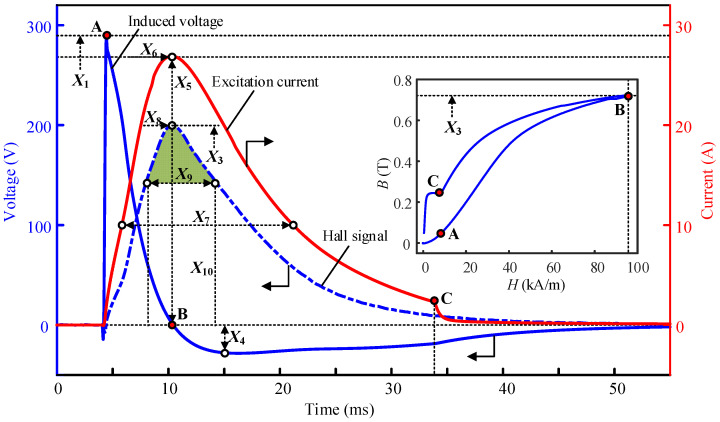
Typical waveforms of measured EM signals.

Figure 3 demonstrates the typical waveforms of the excitation current, *I*_e_(*t*), the induced voltage, *U*_o_(*t*), and the output signal of the Hall sensor, *U_H_*(*t*). The inset shows the calculated magnetization trajectory in the B-H chart. The waveforms in Figure 3 are the source for extracting magnetic features for cable force characterization. A total of ten candidate parameters are selected, and their definitions can be seen from the markers in Figure 3. The symbols and physical meaning of the candidate parameters are listed in Table 2.

**Figure 4:**
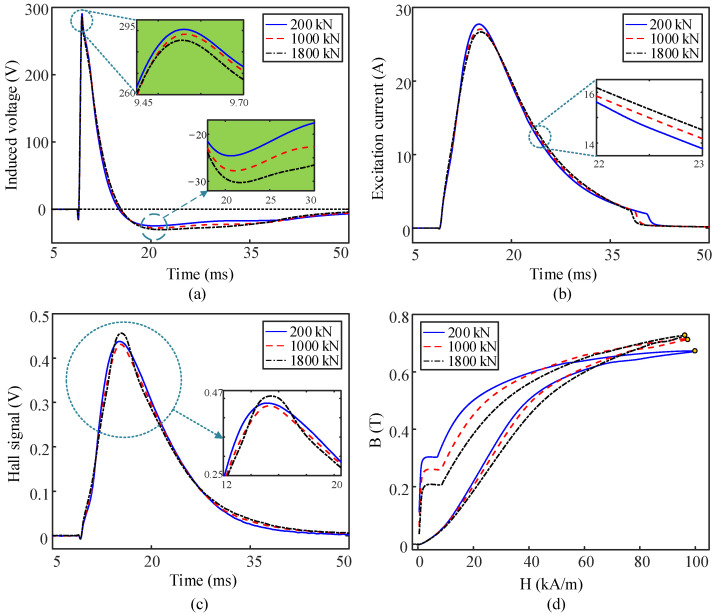
(**a**–**d**) Typical waveforms of the induced voltage, excitation current, output signal of Hall sensor, and B-H curve obtained under different cable forces, respectively.

Figure 4 shows the EM signals measured under the condition of different cable forces. For each case of cable force, five repeat EM tests were performed. The ratio of standard deviation to the mean value of the data (referred to as the coefficient of variation in Table 2) is used as an indicator to evaluate the stability of the EM device in measuring the ten candidate parameters. The data obtained from the case with a cable force of 1200 kN are taken as an example, and the estimated coefficient of variation of all the candidate parameters is lower than 0.5%, indicating the good performance of the developed EM device. It can be observed in Figure 4a–d that the EM signals in different forms are sensitive to the variation of cable force. The performances of the candidate parameters in the quantitative characterization of the cable force will be discussed in Section 4.

## 4. Discussion

Ten candidate parameters selected for the investigation come from different types of EM signals. Some of them are classic and commonly employed by most EM devices such as the parameters of *X*_1_ and *X*_2_. Features with a high sensitivity to cable force, high resolution, and low hysteresis error in cable force measurement are always favored. In addition, linear dependency of the candidate parameter on cable force is of benefit for applications. In this study, the performances of the ten candidate parameters were examined comprehensively for the purpose of seeking new and optimal EM parameters for cable force measurement. To carefully evaluate the performance of the candidate parameters, a scheme of the load–unload cycle with variable steps was performed. In the first stage of loading, the cable was gradually tensioned from 200 kN to around 1000 kN with a step of 200 kN. The force incremental was reduced to 10 kN in the second stage, and the cable was gradually tensioned from 1000 kN to around 1200 kN. In the third stage, from 1200 kN to 1800 kN, the force incremental was restored as 200 kN. The inverse process of loading was applied as the unloading scheme.

**Figure 5:**
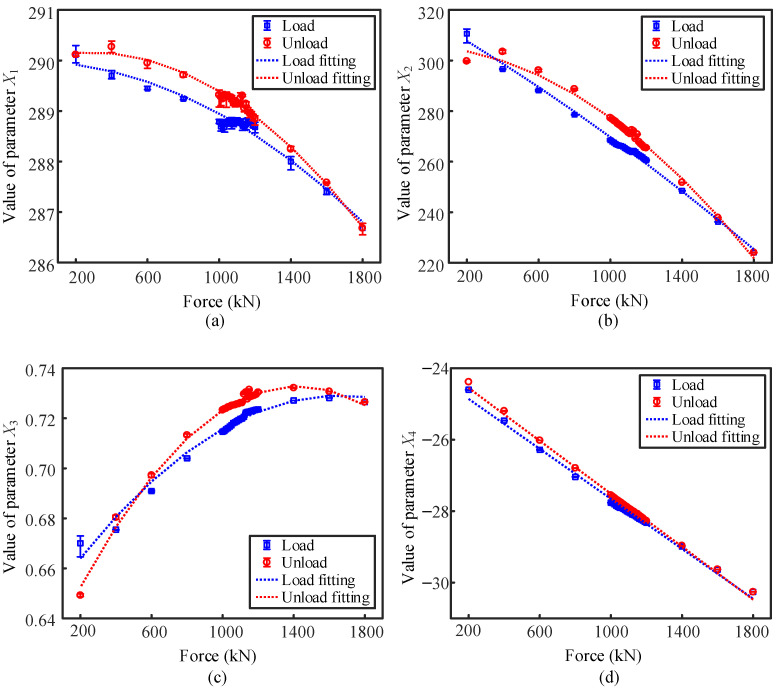
(**a**–**d**) Dependency of *X*_1_, *X*_2_, *X*_3_, and *X*_4_ on the cable force, respectively.

Figure 5 demonstrates the dependencies of the candidate parameters extracted from the induced voltage signal on the cable force. The classic EM parameters of *X*_1_, *X*_2_, and *X*_3_ demonstrated parabolic dependencies on the cable force within the investigated range. Though the goodness of fit of the parabolic equations is higher than 0.95 for all the cases in Figure 5a–c, the calibration curves of *X*_1_, *X*_2_, and *X*_3_ have personalized features. For instance, both the parameters *X*_1_ and *X*_2_ are negatively correlated with the cable force, while an opposite trend can be observed for the dependency of *X*_3_ on the cable force. Monotonic dependencies of *X*_1_ and *X*_2_ on the cable force in the loading direction can be concluded. However, an inflection point appears at the curve of the maximum magnetic induction intensity (*X*_3_) versus cable force in a very high cable force range. Indistinct inflection of the calibration curve also occurs at a very low range of cable force for the parameters of *X*_1_ and *X*_2_ in the unloading process. EM methods with parabolic calibration curves indicate that the sensitivity of the EM method relies on the measured cable force range. For instance, the sensitivity of *X*_3_ on the cable force is significantly decreased in the range beyond 1400 kN as compared with that in the range below 1000 kN. Utilization of non-monotonic curves for the cable force measurement may result in great error caused by varying sensitivity and even multi-valued problems.

Among the investigated candidate parameters of the induced voltage, only the parameter of *X*_4_ (minimum amplitude of the induced voltage) demonstrates the linear correlation with the cable force. In addition, the data points of *X*_4_ measured in the range of 1000–1200 kN are more consistent with the fitting curves than the other parameters. This indicates that the parameter of *X*_4_ may have better resolution in cable force among the four candidate parameters of the induced voltage.

**Figure 6:**
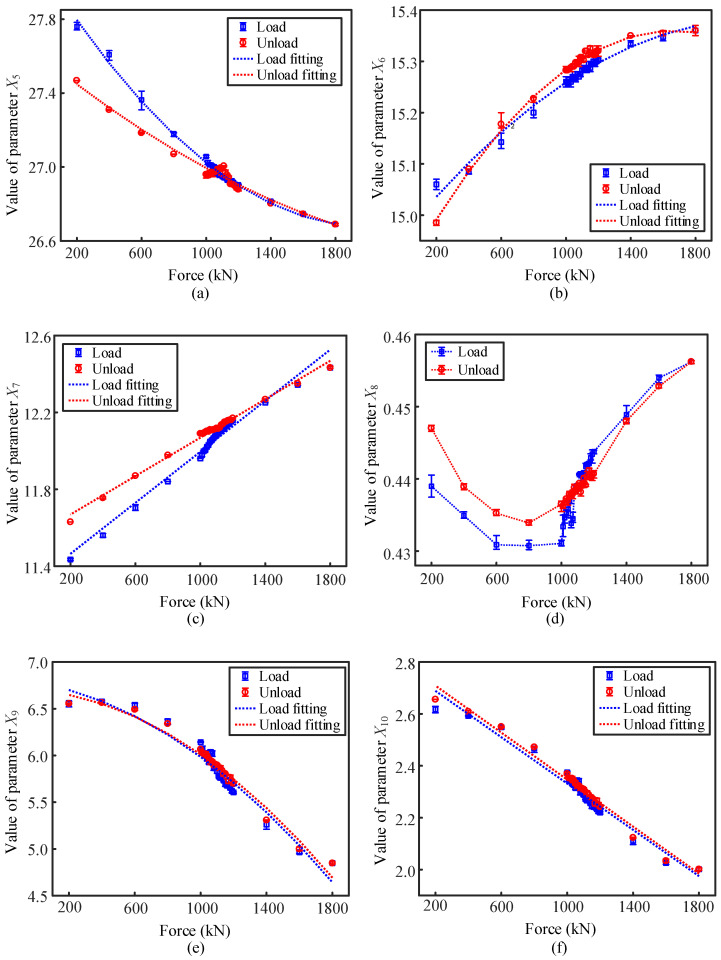
Calibration results about the parameters of (**a**) *X*_5_, (**b**) *X*_6_, (**c**) *X*_7_, (**d**) *X*_8_, (**e**) *X*_9_, and (**f**) *X*_10_.

The performance of the candidate parameters of the excitation current in cable force characterization can be evaluated from the results in Figure 6. A parabolic equation is suitable for describing the relationship between the parameter of *X*_6_^,^ and the cable force. The parameter of *X*_7_ exhibits nearly linear dependency on the cable force in the whole load–unload cycle, although it suffers higher hysteresis error as compared with that of *X*_6_.

Surface magnetic field is not widely used for cable force measurement based on the EM method. In this study, the tangential component of the surface magnetic field is measured by the Hall sensor. The parameters extracted from the signal output by the Hall sensor are used to characterize the cable force, and their performances can be observed in Figure 6d–f. As shown in Figure 6d, the curve of the maximum amplitude of the voltage output by the Hall sensor (*X*_8_) versus cable force is apparently non-monotonic, and a cubic polynomial is needed to accurately fit the measured data points. Thus, the parameter of *X*_8_ will not be discussed later. Both the parameters of *X*_9_ and *X*_10_ are negatively correlated with the cable force. As compared with the parameter of *X*_9_, *X*_10_ is more suitable for cable force measurement due to its linear dependency on the cable force.

**Figure 7:**
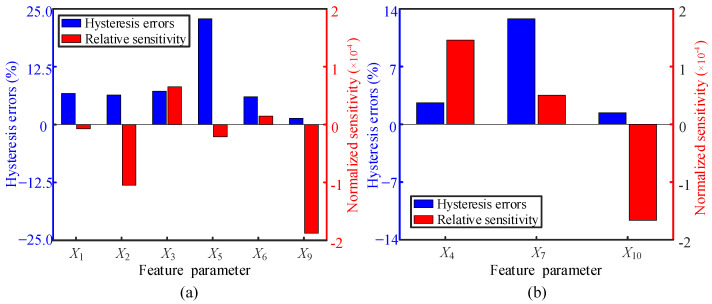
Estimated sensitivity of the parameters on the cable force: (**a**,**b**) results of the parameters belonging to parabolic and linear equations, respectively.

The correlation between the candidate parameters and the cable force can be divided into two categories, which can be displayed by parabolic and linear equations. The information about the fitted equations corresponding to the nine EM parameters is given in Table 3. A comprehensive evaluation of the performance is performed independently of the two categories of candidate parameters. To realize sensitivity comparison between the candidate parameters of different dimensions, normalized sensitivity (α) is introduced as:

(2) $$\alpha =\frac{X_{R}-X_{L}}{X_{L}\cdot \left(F_{\max}-F_{\min}\right)}$$

where *X_R_* and *X_L_* are the value of the parameter at the right endpoint and the left endpoint of the curves in Figure 5 and Figure 6, respectively. *F*_max_ and *F*_min_ represent the upper and lower limits of the applied tensile force, respectively. Hysteresis error of the EM sensor using different parameters is estimated by the equation as follows:

(3) $$\beta =\frac{{\left|X_{\mathrm{load}}-X_{\mathrm{unload}}\right|}_{\max}}{2\left(X_{R}-X_{L}\right)}$$

where *X*_load_ and *X*_unload_ are the value of parameters in the travels of loading and unloading, respectively. For the parameters belonging to parabolic and linear equations, their normalized sensitivity and hysteresis error in cable force characterization are estimated and plotted in Figure 7. When the parabolic equations are employed for EM applications, the parameter of *X*_9_ would be the best for its high sensitivity and low hysteresis error in cable force estimation. EM sensor with high sensitivity and low hysteresis error is more conducive to the practical application of cable force monitoring. Therefore, among the parameters that linearly rely on the cable force, the parameter of *X*_10_ (area under the curve between the two points of 3 dB height of the voltage measured by the Hall sensor) has superior performances in cable force characterization than the parameters of *X*_4_ and *X*_7_.

**Figure 8:**
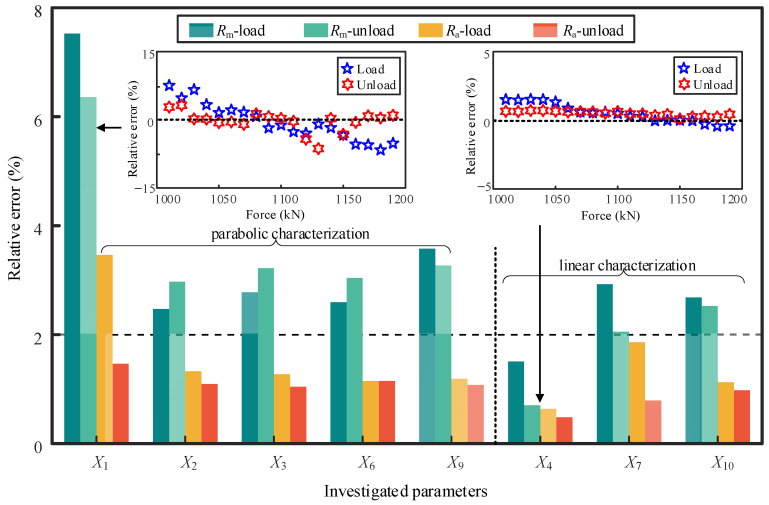
Estimated factor related to the cable force resolution of candidate parameters.

The cable force resolution is an important index for EM devices. In the calibration test, the minimum force increment is about 10 kN, and its corresponding stress is around 5 MPa. The relative error between the measured and the fitted values of candidate parameters is estimated in the range of 1000–1200 kN. Through estimating the maximum relative error (*R*_m_) and the average relative error (*R*_a_) in this particular range, one can evaluate whether the cable force resolution of the individual parameter reaches 10 kN or not. Figure 8 shows the results of *R*_m_ and *R*_a_ for all the screened parameters. Among them, the parameter of *X*_1_, which is commonly used in the EM method, has the worst performance in cable force resolution among the investigated parameters. The parameter of *X*_4_ has the minimum error, so it possesses the best performance in identifying an increment of 10 kN.

It can be concluded from the results in Figure 7 and Figure 8 that the best parameter of EM signal changes is the utilization of different evaluation criteria. To obtain the optimal parameters reasonably, a comprehensive indicator considering the sensitivity, hysteresis error, and cable force resolution is generated as,

(4) $$\delta =\frac{\left|\alpha \right|}{\beta \cdot R_{a}\left|{}_{\;\mathrm{unload}}\right.}$$

where $R_{a}\left|{}_{\mathrm{unload}}\right.$ denotes the estimated average relative error from the unloading process. EM sensors of high sensitivity, low hysteresis error, and high cable force resolution are required for high-quality cable force monitoring. Therefore, the EM parameters with a large value of *δ* are preferred. Table 3 summarizes the estimated values of *δ* related to the performance of candidate parameters in cable force characterization. The frequently used EM parameters (*X*_1_ and *X*_2_) extracted from induced voltage are not the best choice due to their low sensitivity, high hysteresis error, and poor cable force resolution. The peak width at 3 dB height of the voltage measured by the Hall sensor (*X*_9_) demonstrates the best comprehensive performance in cable force characterization among the parameters belongings to the category of the parabola.

In the category of linear characterization, two new parameters demonstrate outstanding characterization ability for cable force, and they are the minimum amplitude of the induced voltage (*X*_4_) and area under the curve between the two points of 3 dB height of the voltage measured by the Hall sensor (*X*_10_). Through comparative analysis and rational evaluation using the proposed comprehensive indicators, the parameters of *X*_4_ and *X*_10_ are recommended for high-performance cable force monitoring. If the parameter of *X*_4_ is employed for cable force monitoring, a secondary coil is required. From the perspective of simplicity in sensor configuring, the parameter of *X*_10_ which can be measured by a small-size Hall device is preferred.

## 5. Conclusions

Cable force monitoring is a significant demand during the maintenance of cable-stayed bridges. The EM method has been proven to be an effective and quantitative means for cable force monitoring. Though many types of EM devices using different EM parameters have been reported, performance comparison analysis is still a difficulty due to the lack of strict comparative experiments and unified evaluation indicators. In this study, based on the EM effect, the Hall sensor and the excitation coil (self-inductance effect) were proposed to possibly substitute the traditional induction coil. Compared with traditional parameters, the new characterized parameters could improve the detection accuracy of the cable force. The main conclusions are the following:

(1) Strict comparative experiments were performed on a cable composed of a seven-wire steel stand to examine the comprehensive capability of cable force measurement. The experimental set-up using a self-developed and miniaturized EM device could synchronously record the excitation pulse current, the voltage induced in the secondary coil, and the surface magnetic field measured by the Hall sensor.
(2) The evaluation method of parameters for cable force detection was proposed. A total of ten EM parameters were investigated by evaluating their dependency on the cable force and sensitivity, hysteresis error, and resolution in cable force measurement. The comprehensive indicator of *δ* considering the sensitivity, hysteresis error, and cable force resolution was feasible for filtering the best EM parameters.
(3) In the category of parabolic characterization, the parameter of *X*_9_ (peak width at 3 dB height of the voltage measured by the Hall sensor) was the optimal one. Compared with traditional parameters, it was recommended for high-performance cable force monitoring. Among the parameters (*X*_4_, *X*_7_, *X*_10_) demonstrating linear correlation with the cable force, the parameters of *X*_4_ (minimum amplitude of the induced voltage) and *X*_10_ (area under the curve between the two points of 3 dB height of the voltage measured by the Hall sensor) were recommended for high-performance cable force monitoring. Both the parameters of *X*_4_ and *X*_10_ have not been reported yet, and the latter could be measured using a small-size Hall device without the requirement of a secondary coil.

The proposed comprehensive indicator can be employed as a criterion for performance comparison between EM devices and also an inspirational tool for optimally selecting EM parameters extracted from the abundant signals related to cable magnetization. In the future, the EM parameters selection method presented in this work will be applied to cables of different structures.

## Figures and Tables

**Table 1:** Technical parameters of the coil sensor and the Hall sensor.

**Parameters**	**Excitation/Primary Coil**	Sensing/Secondary Coil
Inner diameter, mm	130	100
Outer diameter, mm	185	126
Height, mm	140	35
Number of coil turns	7200	336
Number of layers	4	4
Number of conductors in a single cable	12	7
Outer diameter of cable, mm	6.8	3.2
Connection mode between coils of each layer	In parallel	In series

**Table 2:** Feature parameters extracted from the typically measured EM signals.

Signal Type	Indicators	Descriptions	Variation Coefficients
Induced voltage, *U*_o_(*t*)	*X*_1_ (V)	Maximum amplitude of the induced voltage	0.026%
	*X*_2_ (V·ms)	Integral value of the induced voltage curve	0.421%
	*X*_3_ (T)	The maximum value of estimated magnetic induction intensity	0.294%
	*X*_4_ (V)	Minimum amplitude of the induced voltage	0.021%
Excitation current, *Ie*(*t*)	*X*_5_ (A)	Maximum amplitude of the excitation current	0.035%
	*X*_6_ (ms)	Peak position of the maximum amplitude of the excitation current	0.040%
	*X*_7_ (ms)	Full width at half maxima of excitation current	0.022%
Hall signal, *U_H_*(*t*)	*X*_8_ (V)	Maximum amplitude of voltage measured by Hall sensor	0.218%
	*X*_9_ (ms)	Peak width at 3 dB height of voltage measured by Hall sensor	0.228%
	*X*_10_ (V·ms)	Area under the curve between the two points of 3 dB height of the voltage measured by Hall sensor	0.182%

**Table 3:** Evaluation factors related to the performance of candidate parameters.

Parameters	Sensitivity,|α|	Hysteresis Error, *β*	Indicator, *δ*	Fitted Equation	
				Load Process	Unload Process
*X*_1_	−0.07	6.729	0.0075	*y* = −9.11 × 10^−7^*x*^2^ − 1.23 × 10^−4^*x* + 289.98	*y* = −1.51 × 10^−6^*x*^2^ + 8.55 × 10^−4^*x* + 290.04
*X*_2_	−1.046	6.382	0.1469	*y* = −4.96 × 10^−6^*x*^2^ − 4.16 × 10^−2^*x* + 316.26	*y* = −2.28 × 10^−5^*x*^2^ − 5.48 × 10^−3^*x* + 305.69
*X*_3_	0.653	7.190	0.0927	*y* = −3.03 × 10^−8^*x +* 1.01 × 10^−4^*x* + 0.64	*y* = −5.37 × 10^−8^*x +* 1.53 × 10^−4^*x* + 0.62
*X*_6_	0.145	6.012	0.0220	*y* = −8.83 × 10^−8^*x*^2^ + 3.85 × 10^−4^*x* + 14.96	*y* = −1.71 × 10^−7^*x*^2^ + 5.71 × 10^−4^*x* + 14.88
*X*_9_	−1.879	1.335	1.2802	*y* = −4.89 × 10^−7^*x*^2^ − 3.09 × 10^−4^*x* + 6.79	*y* = −5.33 × 10^−7^*x*^2^ − 1.57 × 10^−4^*x* + 6.70
*X*_4_	1.457	2.606	1.1859	*y* = −3.50 × 10^−3^*x* − 24.16	*y* = −3.70 × 10^−3^*x* − 23.81
*X*_7_	0.504	12.792	0.1423	*y* = 6.64 × 10^−4^*x +* 11.33	*y* = 5 × 10^−4^*x +* 11.57
*X*_10_	−1.665	1.404	1.2121	*y* = −4.46 × 10^−4^*x* + 2.78	*y* = −4.53 × 10^−4^*x* + 2.80

## References

[1] Hu D., Guo Y., Chen X., Zhang C. (2017). Cable force health monitoring of Tongwamen bridge based on fiber Bragg grating. Appl. Sci..

[2] Zhang L., Qiu G., Chen Z. (2021). Structural health monitoring methods of cables in cable-stayed bridge: A review. Measurement.

[3] Zhang X., Lu Y., Cao M., Li S., Sumarac D., Wang Z. (2022). Instantaneous identification of tension in bridge cables using synchrosqueezing wave-packet transform of acceleration responses. Struct. Infrastruct. Eng..

[4] Cappello C., Zonta D., Laasri H.A., Glisic B., Wang M. (2018). Calibration of elasto-magnetic sensors on in-service cable-stayed bridges for stress monitoring. Sensors.

[5] Liu Z., Liua S., Xie C., Bai G. (2021). Non-invasive force measurement based on magneto-elastic effect for steel wire ropes. IEEE Sens. J..

[6] Ren L., Xiu C., Li H., Lu Y., Wang J., Yao X. (2018). Development of elasto-magnetic (EM) sensor for monitoring cable tension using an innovative ratio measurement method. Smart Mater. Struct..

[7] Ceballos M.A., Prato C.A. (2008). Determination of the axial force on stay cables accounting for their bending stiffness and rotational end restraints by free vibration tests. J. Sound Vib..

[8] Klier T., Míčka T., Polák M., Plachý T., Hedvábný M., Jelínek R., Bláha F. (2018). Application of the Modified Magnetoelastic Method and an Analysis of the Magnetic Field. Acta Polytech. CTU Proc..

[9] Zhang S., Zhou J., Zhou Y., Zhang H., Chen J. (2019). Cable tension monitoring based on the elasto-magnetic effect and the self-induction phenomenon. Materials.

[10] Abdel-Jaber H., Glisic B. (2019). Monitoring of prestressing forces in prestressed concrete structures—An overview. Struct. Control. Health Monit..

[11] Wang M.L., Chen Z.L., Koontz S.S., Lloyd G.M. (2000). Magnetoelastic permeability measurement for stress monitoring in steel tendons and cables. Proc. SPIE-Int. Soc. Opt. Eng..

[12] Wang M.L., Wang G., Zhao Y. (2005). Application of EM stress sensors in large steel cables. Smart Structures and Materials.

[13] Kim W.K., Kim J., Park J., Kim J.W., Park S. (2022). Verification of Tensile Force Estimation Method for Temporary Steel Rods of FCM Bridges Based on Area of Magnetic Hysteresis Curve Using Embedded Elasto-Magnetic Sensor. Sensors.

[14] Liu X., Wu D., He C., Feng H., Wu B. (2018). Comparison of AC and pulsed magnetization-based elasto-magnetic methods for tensile force measurement in steel strand. Measurement.

[15] Zhang R., Duan Y., Zhao Y., He X. (2018). Temperature compensation of elasto-magneto-electric (EME) sensors in cable force monitoring using BP neural network. Sensors.

[16] Duan Y.F., Zhang R., Dong C.Z., Luo Y.Z., Or W.S., Zhao Y., Fan K.Q. (2016). Development of elasto-magneto-electric (EME) sensor for in-service cable force monitoring. Int. J. Struct. Stab. Dyn..

[17] Hu X., Duan Y., Luo Y., Yun C. (2021). Development of Elasto-Magneto-Electric Sensors for Total-Stress in Large-Diameter Cables. EASEC16.

[18] Feng H., Liu X., Wu B., Wu D., Zhang X., He C. (2019). Temperature-insensitive cable tension monitoring during the construction of a cable-stayed bridge with a custom-developed pulse elasto-magnetic instrument. Struct. Health Monit..

